# Army Medical Board

**Published:** 1843-06

**Authors:** 


### ARMY MEDICAL BOARD.

A Board for the examination of such assistant surgeons as may be ordered to appear before it, and such applicants for appointment in the medical staff of the army as may be invited to attend, will convene in the city of New York on the first of July. The Board will consist of Surgeons Thomas G. Mower, Henry A. Stinnecke, Charles S. Tripler, and Assistant Surgeon, J. B. Wright, as supernumerary and recorder. C.

